# Discovering the neuronal dynamics in major depressive disorder using Hidden Markov Model

**DOI:** 10.3389/fnhum.2023.1197613

**Published:** 2023-06-29

**Authors:** Wenhao Jiang, Shihang Ding, Cong Xu, Huihuang Ke, Hongjian Bo, Tiejun Zhao, Lin Ma, Haifeng Li

**Affiliations:** ^1^Faculty of Computing, Harbin Institute of Technology, Harbin, China; ^2^Shenzhen Academy of Aerospace Technology, Shenzhen, China

**Keywords:** electroencephalogram, major depressive disorder, Hidden Markov Model, neuronal dynamics, temporal encoding

## Abstract

**Introduction:**

Major Depressive Disorder (MDD) is a leading cause of worldwide disability, and standard clinical treatments have limitations due to the absence of neurological evidence. Electroencephalography (EEG) monitoring is an effective method for recording neural activities and can provide electroneurophysiological evidence of MDD.

**Methods:**

In this work, we proposed a probabilistic graphical model for neural dynamics decoding on MDD patients and healthy controls (HC), utilizing the Hidden Markov Model with Multivariate Autoregressive observation (HMM-MAR). We testified the model on the MODMA dataset, which contains resting-state and task-state EEG data from 53 participants, including 24 individuals with MDD and 29 HC.

**Results:**

The experimental results suggest that the state time courses generated by the proposed model could regress the Patient Health Questionnaire-9 (PHQ-9) score of the participants and reveal differences between the MDD and HC groups. Meanwhile, the Markov property was observed in the neuronal dynamics of participants presented with sad face stimuli. Coherence analysis and power spectrum estimation demonstrate consistent results with the previous studies on MDD.

**Discussion:**

In conclusion, the proposed HMM-MAR model has revealed its potential capability to capture the neuronal dynamics from EEG signals and interpret brain disease pathogenesis from the perspective of state transition. Compared with the previous machine-learning or deep-learning-based studies, which regarded the decoding model as a black box, this work has its superiority in the spatiotemporal pattern interpretability by utilizing the Hidden Markov Model.

## 1. Introduction

Major depressive disorder (MDD), which is known to be characterized by persistent sadness, irritability, frustration, and restlessness, is among the ten leading causes of worldwide disability-adjusted life years according to the World Health Organization (Mathers et al., 2006). The absence of neurological evidence has posed a great challenge to the current standard clinical treatment, which involves medications and psychotherapy minu1pt (Olfson et al., 2016). Pursuant to past studies, one-third of MDD patients failed to induce remission after adequate antidepressant trials (Rush et al., 2009). The benefits of studying the neurological markers of MDD are three-fold: (i) Early stage MDD diagnosis. By identifying neurobiological markers that are quantifiable and precisely locatable, it is possible to expedite and enhance the accuracy of early stage depression diagnosis, thus preventing misdiagnosis and minimizing treatment delay (Olbrich and Arns, 2013; Mahato and Paul, 2019); (ii) Device-based intervention. The specification of neurobiological markers for depression can facilitate effective interventions targeting specific brain regions or circuits, such as electroconvulsive therapy (ECT), repetitive transcranial magnetic stimulation (rTMS), transcranial direct current stimulation (tDCS), vagus nerve stimulation (VNS), magnetic seizure therapy (MST) and deep brain stimulation (DBS) (Caldieraro and Cassano, 2019). (iii) Precision medicine. The identification of neurobiological markers for MDD can pave way for the targeted neuropharmacological treatment development, thereby enabling more precise pharmacotherapy (Manchia et al., 2020).

However, uncovering the neurobiological markers for MDD is a complex and challenging task. Firstly, the high degree of heterogeneity in MDD, including diverse symptom profiles, severity levels, and underlying neurobiological mechanisms, raises difficulties in the identification of reliable neurobiological markers. Secondly, the transition patterns among brain activity states during rest or cognitive tasks in MDD patients remain uncertain, making it difficult to accurately characterize the temporal features of neural activities. Nevertheless, neural activities of the brain are conveyed through electrophysiological signals, which are fast-changing signals that require analysis at a subtle temporal granularity. Therefore, a methodology is needed to analyze the patterns of neural activities and their transformation processes based on neural recordings from clinically diagnosed MDD patients, which are feasible to deploy on a large scale in clinical settings. Until recently, the broad deployment of electroencephalographic (EEG) in MDD treatment and the development of advanced processing and analyzing methods have paved the way for an interdisciplinary solution to this dilemma. Since EEG measures the electrical activities of large and synchronously firing populations of neurons via the electrodes placed on the scalp, long-term resting-state EEG monitoring, as well as short-term task-related EEG signals of the MDD brain is conducive to providing eletroneurophysiological evidence of MDD.

Numerous studies have utilized machine learning or deep learning algorithms to extract high-dimensional features from EEG signals and classify the EEGs of patients with MDD and healthy controls (HC). In the study conducted by Duan et al. (2020), EEG signals were acquired from 32 participants, comprising 16 MDD and 16 HC individuals, and subject to frequency-domain functional connectivity analysis. The functional connectivity matrix obtained was subjected to classification using support vector machine (SVM) and convolutional neural network (CNN) algorithms, yielding a testing set accuracy of 94.13%. In another functional brain network (FBN) study for MDD analysis (Zhang et al., 2021), the phase lag index (PLI) was calculated to construct a functional connection matrix. Alterations of brain synchronization were discovered on an EEG dataset consisting of 48 subjects (24 MDD, 24 HC). A network decomposition model based on Improved Empirical Mode Decomposition (EMD) was proposed for the time-frequency analysis of FBN on MDD subjects (Shao et al., 2021). By constructing FBN on different intrinsic mode functions (IMF), the authors performed the time-frequency analysis of brain function connections and validated the proposed model on 128-channel EEG signals. Despite achieving high accuracy in identifying MDD/HC and discovering statistically significant brain functional connectivity abnormalities in MDD patients, the aforementioned studies have overlooked the dynamic variability of brain functional networks and neglected the dynamic transitions between different network patterns in MDD patients.

In order to address this issue, the Hidden Markov Model (HMM) was employed to represent brain activities as a sequence of discrete brain states in temporal scales derived directly from the data, offering significant improvements over the sliding window approach in previous studies (Vidaurre et al., 2016, 2017; Stevner et al., 2019). Several previous studies have demonstrated the capability of the HMM to capture the dynamic behavior of brain activity in short timescales (Vidaurre et al., 2018; Stevner et al., 2019). Notably, recent research utilizing magnetoencephalography (MEG) has shown that the HMM can accurately capture brain activity in a resting state in intervals as brief as 100 ms (Quinn et al., 2018). Furthermore, the HMM has been proposed as a useful tool to explore the reconfiguration of whole-brain dynamics associated with ASP (Lin et al., 2022) and MDD (Wang et al., 2020), and has the potential to provide a comprehensive description of the brain dynamics in short timescales.

In this study, we employed the HMM with multivariate autoregressive observation (HMM-MAR) model (Vidaurre et al., 2016) on a 128-channel task-related EEG dataset (Cai et al., 2022), consisting of 53 subjects with 24 diagnosed with MDD and 29 HC controls. In order to assess the fidelity of the HMM model in capturing the EEG state transition patterns of MDD/HC participants, we employed the proposed model to decode the PHQ-9 scores of the participants. Specifically, we utilized the EEG signals of the participants in response to presented stimuli and trained an HMM-MAR model to perform a regression analysis on the participants’ Patient Health Questionnaire-9 (PHQ-9) scores. The parameters of the model were updated using the variational Bayesian inference. The outcoming results suggest that the obtained state time series can effectively characterize neural dynamic features. Furthermore, coherence analysis and power spectrum estimation reveal significant differences in the state time series of MDD and HC subjects across different emotional stimuli, as observed in the corresponding EEG signals. These differences are consistent with previous research findings on depression neural circuits and brain network analysis, highlighting the potential of utilizing HMM models to characterize neural dynamic features for clinical applications.

Generally, the major work contributions are: **i)** Distinct patterns of brain activity state transitions in response to different emotional stimuli were identified in patients diagnosed with MDD in comparison to HC. Brain connectivity analyses of the corresponding patterns to these stimuli were conducted in both groups. **ii)** The brain activity state transition patterns in response to sadness-related stimuli had the best fit with the scores on the PHQ-9 among all stimuli presented to participants in the study. These patterns are therefore more likely to indicate a risk for anxiety and MDD in response to sadness-related stimuli. **iii)** The outcoming results suggest that our finding introduces a novel approach to the diagnosis of MDD, and shows the potential to be integrated into the clinical treatment workflow.

## 2. Materials and methods

### 2.1. MODMA EEG dataset

We used the MODMA dataset,^1^ from Lanzhou University (Hu et al., 2017; Li et al., 2018), as the research subject. This dataset collected resting-state and task-state electroencephalography (EEG) data from 53 participants (Age: 18–52, Female: 37.7%), including 24 individuals with major depressive disorder (MDD) and 29 healthy controls (HC). All participants who were classified into the MDD group were diagnosed by experienced clinical psychologists according to the DSM-IV inclusion criteria. Scalp EEG was recorded using the EGI series 128-channel EEG acquisition system manufactured by Electrical Geodesics Inc. All the participants went through psychological assessment scales, such as PHQ-9, Generalized Anxiety Disorder (GAD-7), Social Support Research Scale (SSRS), Life Event Scale (LES), and Childhood Trauma Questionnaire (CTQ-SF). Inclusion criteria for MDD group included meeting the diagnostic criteria of the Mini-International Neuropsychiatric Interview (MINI) for depression, scoring greater than or equal to 5 on the PHQ-9, and having abstained from psychotropic drug treatment for at least 2 weeks prior to the study.

In this experiment, participants were asked to complete an experimental task using a modified dot-probe paradigm while seated 60 cm away from a 17′′ monitor with 1280 × 1024 resolution and a 60 Hz refresh rate. Before the formal experiment, participants completed 10 practice trials to become familiar with the task. During the experiment, participants viewed emotional face pairs and identified the location of a dot by pressing a button on a reaction box without moving their bodies or eyes. The experiment consisted of three blocks, each with 160 trials of fear-neutral, sad-neutral, and happy-neutral pairs. Each trial lasted approximately 1.6 s and the entire experiment took around 25 min. The experiment settings were described in detail by Cai et al. (2022).

### 2.2. Systematic framework

**FIGURE 1:**
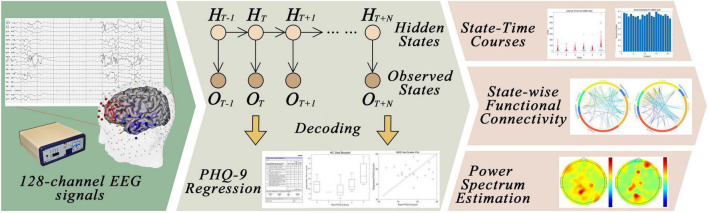
Schematic workflow of the proposed Hidden Markov Model (HMM).

The workflow for the proposed MDD neurological state analysis framework is shown in Figure 1. The system comprises three major parts: (i) The preprocessing of EEG signals; (ii) The implementation of the HMM-MAR model, whose parameters are updated using the variational Bayesian inference; (iii) The analysis of state-time courses, state-wise functional connectivity, power spectrum estimation, and coherence analysis. The detailed descriptions of the aforementioned parts are listed below.

### 2.3. EEG preprocessing

The EEG signal preprocessing pipeline is as follows: Initially, the raw signal undergoes a bad segment removal process. It is then subjected to band-pass filtering in the frequency range of 0.5–70 Hz, in addition to notch filtering at 50 Hz to eliminate artifacts due to motion, noise, and powerline interference. Next, the EEG is re-referenced to the average electrode, followed by an independent component analysis (ICA) procedure (Makeig et al., 1995). The ICA removes components that are influenced by eye movement, motion, heartbeat, and bad channels, which results in preprocessed EEG signals. The aforementioned data preprocessing steps were performed using the EEGLAB toolbox (Delorme and Makeig, 2004), with the ICLabel plugin (Pion-Tonachini et al., 2019) utilized for ICA component selection. We manually selected and excluded the data with significant artifacts and bad channel influence. A total of 43 eligible participants were included in the study’s EEG dataset out of the 53 initially recruited individuals. The preprocessed EEG segments ranging from 200 ms pre-stimulus to 800 ms post-stimulus for each participant were utilized as input, and subsequently entered into the neural dynamic decoding model.

### 2.4. HMM-MAR model

The Hidden Markov Model (HMM) is a probabilistic graphical model that is commonly used to model time-series data. It is designed to describe a Markov process with a hidden, unknown variable that depends only on the current state and not any previous state. The HMM is comprised of two basic components: a state sequence and an observation sequence. The state sequence is a sequence of hidden states in a time series that transition based on certain rules, while the observation sequence refers to the data observed at each time point, which may or may not be related to the state sequence. The basic assumption of the HMM is that the observation sequence is generated only by the corresponding state at each time point. To extract features from time-series data and model multivariate time-series data, Fox et al. (2009) proposed the Beta-Process Auto-Regressive Hidden Markov Model (BP-AR-HMM), which combines the traditional HMM with the autoregressive model (AR) and Beta Process initialization. This model can automatically extract features from time-series data and model multivariate time-series. Building on the BP-AR-HMM model, Vidaurre et al. (2017) proposed a full probabilistic representation of the multivariate autoregressive model and used variational Bayesian inference to estimate the model parameters.

To be specific, we denote the multichannel EEG signal as *O*_*t*_ ∈ ℝ^*N*^, and the hidden states as *H_t_*, where *t* 1, ⋯, *T*. Assuming Gaussian noise, the observation model could be depicted as:


(1) $$O_{t}|H_{t}=k∼𝒩\,\left(\sum_{l\in \rho }y_{t-l}\,W_{l}^{\left(k\right)},\sum^{\left(k\right)}\right),$$

where ρ presents the lags of the MAR model, $W_{l}^{\left(k\right)}$ presents the *k*-th state AR coefficient matrix, and ∑^(*k*)^ the diagonal noise covariance matrix. The inverse of ∑^(*k*)^ could be modeled with a Wishart distribution, and Gamma distribution if diagonal.


(2) $$\sum_{}^{\;{\left(k\right)}^{-1}}∼Wishart({ı}_{0},\;{ℬ}_{0}),$$

(3) $$\varpi_{i\,i}^{\left(k\right)}∼G\,a\,m\,m\,a\,\left({ı}_{0},{ℬ}_{0}\right),$$

The probability representation of the Markov dynamics can be depicted as:


(4) $$P\left(H_{t}=k_{1}|H_{t-1}=k_{2}\right)=\Theta_{k_{1}\,k_{2}},$$

(5) $$P\left(H_{1}=k\right)=\eta_{k}$$

where Θ_*k*_1_*k*_2__ and η_*k*_ are the HMM-MAR model parameters to be inferred, and are modeled with Dirichlet distribution:


(6) $$\Theta_{k}∼D\,i\,r\,\left(\nu_{0}\right)$$

(7) $$\eta ∼D\,i\,r\,\left(\xi_{0}\right)$$

where ν_0_ and ξ_0_ are the parameters inferred via the HMM-MAR training process.

The Bayesian hierarchy governing the model’s parameters was established from Eqs. (1∼7). The variational Bayes method, which assumes additional factorizations in the parameter space and requires conjugate prior distributions (Wang and Fan, 2019), was implemented for parameter inference. This approach utilizes an iterative algorithm that operates on one group of parameters at a time to minimize the free energy, a quantity that is useful for monitoring and model selection. The algorithm seeks to minimize the cost function known as free energy, which is computed as the sum of the model’s average log-likelihood, the Kullback-Leibler divergence (KL-divergence) between the actual and factorized distributions, and the negative entropy of the factorized distribution. The variational approach involved alternating between a (variational) E-step and M-step. The E-step involved estimating the probabilities of the hidden states, while the M-step involved estimating the model parameters. This statistic is described in detail in the previous studies (Rezek et al., 2005), while others (Foti et al., 2014) provide further mathematical elaborations on the algorithm and cost function.

In the model implementation, we followed previous research experiences (Tao et al., 2022) and specified the number of hidden states as 6 and the order of the MAR model as 3 in the HMM framework. Additionally, the stochastic inference was performed using a batch size of 15, forget rate of 0.7, and a base weight of 0.9. The experiments were implemented on a high-performance computing unit equipped with a 48-core Intel Xeon E5-2696 processor and 192GB of RAM.

## 3. Results

### 3.1. Regression results for PHQ-9 score

**FIGURE 2:**
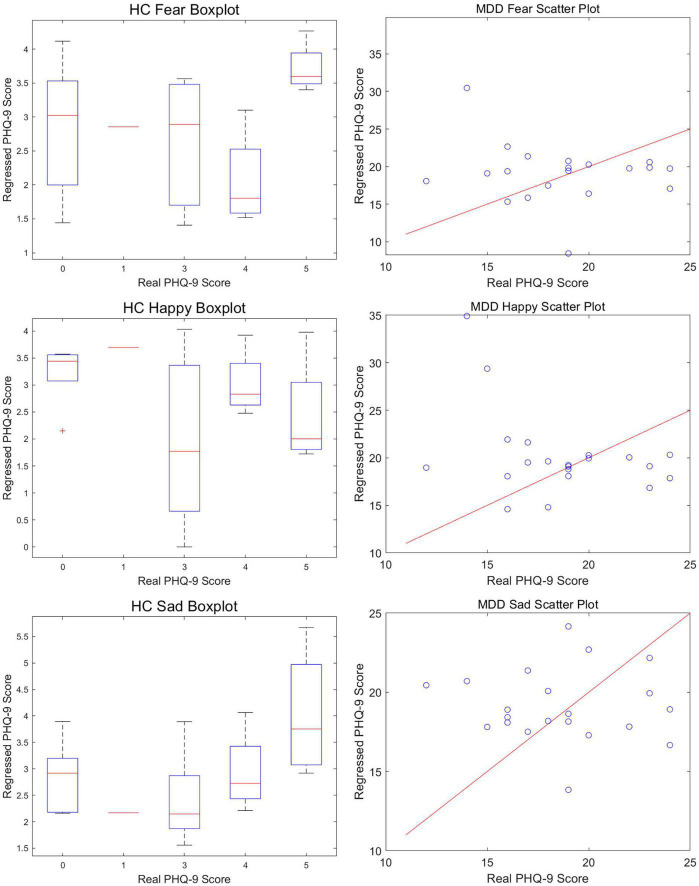
Regression results regarding the PHQ-9 scores.

We partitioned the preprocessed EEG data of all participants into six groups, consisting of MDD patients presented with happy, sad, and fearful stimuli, and healthy controls presented with happy, sad, and fearful stimuli. We extracted EEG segments ranging from 200 ms before stimulus onset to 800 ms post-stimulus onset from each group and concatenated all trials of all participants within each group to form six long sequences. We then applied the HMM-MAR model to each sequence for neural dynamic activity modeling. To verify the decoding ability of the HMM-MAR model for the dynamic neural activity of MDD patients and healthy controls in response to the three types of stimuli, we regressed the state time series obtained from the model for each of the six groups against the PHQ-9 scores of each participant within each group, due to its suitability for measuring the severity of depressive symptoms. The regression results are shown in Figure 2 and Table 1.

**TABLE 1:** Comparisons among six groups on PHQ-9 score regression.

	H_F	H_H	H_S	M_F	M_H	M_S
MAE	1.59	2.04	1.43	4.85	4.15	3.33
MSE	3.55	5.49	2.96	52.59	42.08	16.30
RMSE	1.88	2.34	1.72	7.25	6.49	4.04

H_F, HC group with fear face stimuli; H_H, HC group with happy face stimuli; H_S, HC group with sad face stimuli; M_F, MDD group with fear face stimuli; M_H, MDD group with happy face stimuli; M_S, MDD group with sad face stimuli; MAE, Mean Absolute Error; MSE, Mean Square Error; RMSE, Root Mean Square Error.

In order to better demonstrate the results. Due to the relatively concentrated PHQ-9 scores of the healthy control group and the scattered scores of the MDD group, we used a box plot to depict the relationship between the actual PHQ-9 scores of the healthy control group and the estimated values obtained through state time series regression and used a scatter plot to describe the MDD group. The comparison details were illustrated in Figure 2.

In Table 1, we quantitatively compared the regression performance of the PHQ-9 scores for the six experimental and control groups using the MAE, MSE, and RMSE. From Figure 2 and Table 1, we observed that regardless of the MDD or HC group, using the HMM-MAR model to obtain the state time series of participants’ brain signals when exposed to sad face stimuli resulted in the best regression of participants’ PHQ-9 scores. This indicates that the HMM-MAR model is more capable of decoding the neural dynamics of the brain when processing sad emotions, and the brain state under sad stimuli presents a more obvious Markov property.

### 3.2. State-time courses analysis

**FIGURE 3:**
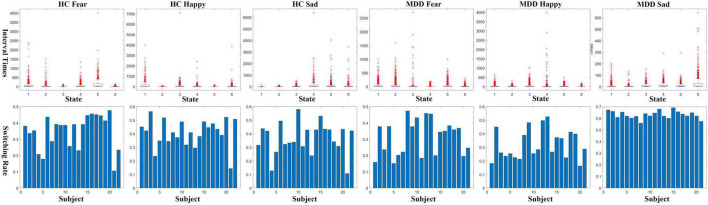
Interval times **(upper row)** and switching rates **(lower row)** of the decoded state-time courses.

To examine the inferred states, global temporal statistics were calculated and analyzed. In this work, we calculated the interval times of each HMM state in each of the six experimental and control groups. The switching rates of each subject for each group were also calculated and compared, shown in Figure 3. The interval times for each group are composed of six vectors indicating the number of time points between state visits. The results in Figure 3 show that in the six experimental groups, the interval time distributions of the six states are relatively evenly distributed. Although some states have more outliers, there is no dominant state in any of the groups. This demonstrates that the neural dynamic state time series decoded by the HMM-MAR model is evenly distributed. Meanwhile, we also provided measurements of the state switching rate for each subject, which can be understood as a measure of stability per subject. The results presented in Figure 3 reveal that the switching rates among subjects vary considerably in response to fearful or happy facial stimuli, whereas such rates remain relatively consistent when MDD subjects are presented with sad facial stimuli. These findings align with the conclusions drawn in the previous section.

### 3.3. State-wise neural dynamic

**FIGURE 4:**
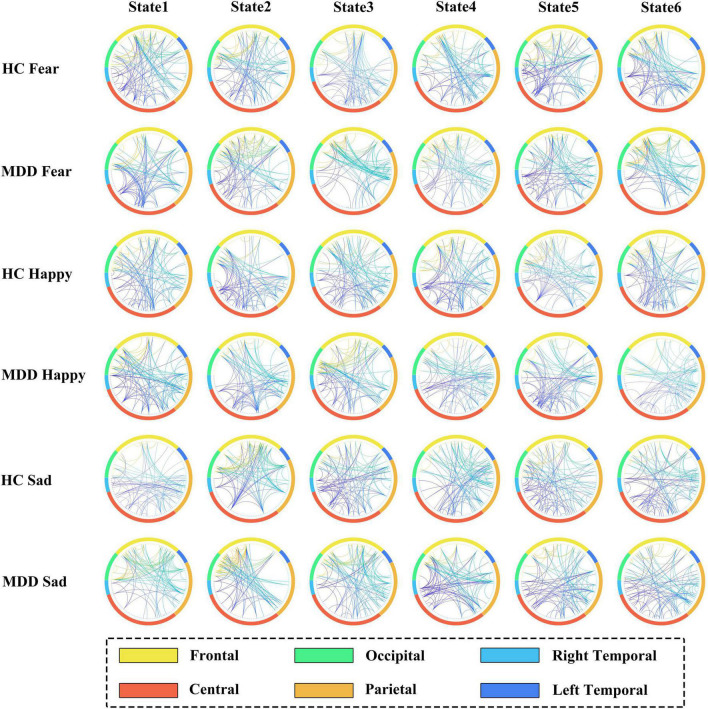
Channel-wise coherence connectivity for each HMM states.

Our study performed a frequency domain analysis of the states (states 1-6) inferred by the HMM-MAR model in the MDD and HC control groups and computed the coherence between channels. To partition the 128 EGI-Geodesics EEG cap electrodes into six regions, we followed the method proposed by (Sleeping) et al. Specifically, we divided the electrodes into six regions, namely Frontal, Central, Right Temporal, Left Temporal, Parietal, and Occipital, based on their layout. The coherence between EEG channels was calculated for three distinct emotional facial stimuli in the MDD and HC control groups, we preserved the top 1% coherences and generated a connectivity map as presented in Figure 4.

The following conclusions can be drawn from Figure 4. For both the MDD and HC groups presented with sad facial stimuli, the coherence between the frontal electrode and other electrode regions is significantly reduced in the MDD group, indicating weakened channel connectivity. This may be related to frontal lobe dysfunction in MDD patients, resulting in different brain connectivity patterns induced by sad facial stimuli compared to HC. There was no significant difference between the MDD and HC groups presented with fearful facial stimuli, while in the experiment with happy facial stimuli, MDD patients showed differences in connectivity patterns in the right temporal and occipital regions compared to HC. This may be related to the different brain activity patterns of MDD patients in response to happy stimuli.

### 3.4. Power spectrum

**FIGURE 5:**
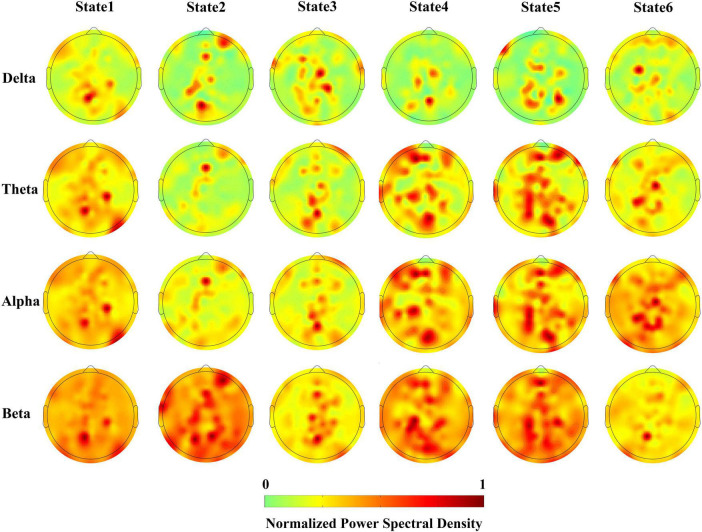
Power spectrum topology for each HMM states.

We adopted a parametric autoregressive modeling approach, namely the MAR, to estimate the power spectra of different neural dynamic transitions observed in the MDD and HC control groups during three distinct stimuli. It is noteworthy that the MAR model for power spectrum estimation is not the same as that used in the HMM-MAR model. An MAR model with a model order of 3 was trained separately in the process. Specifically, we computed the power spectra of the six states in six control groups (MDD and HC) across Delta (0–4 Hz), Theta (4–8 Hz), Alpha (8–12 Hz), and Beta (12–40 Hz) frequency bands. To accommodate space limitations, we present the scalp maps of the power spectra estimation, with each map normalized to 0∼1, in response to the sad emotional facial stimulus for the MDD group in Figure 5.

Utilizing power spectra, we can approximate the level of activation of brain regions corresponding to scalp EEG electrodes. From Figure 5, the following observations can be made: Firstly, in terms of distribution, when presented with sad facial stimuli, the activation regions in MDD patients are mainly distributed in the parietal and frontal lobes. In the vertical direction, the power of the electrode corresponding to the parietal lobe in the delta frequency band is higher, indicating stronger activation, while in the theta and alpha frequency bands, activation is observed in both the parietal and frontal lobes. Secondly, different neural dynamic patterns decoded by HMM-MAR exhibit differences in activation patterns. In the horizontal direction, differences in activation regions for states 1 to 6 could be observed in all frequency bands, indicating the presence of Markovian properties in the brain activation patterns of MDD patients when presented with sad stimuli.

## 4. Discussion

This study aimed to decode the neural dynamics of EEG signals in response to happy, sad, and fearful facial stimuli in both clinically diagnosed MDD patients and healthy controls, using the HMM-MAR model. Specifically, we trained the HMM-MAR on the MODMA dataset using two groups of samples, MDD and HC, exposed to stimuli of happiness, fear, and sadness, respectively, to obtain the state time series. To validate the accuracy of the model, we designed a regression task that utilized the state time series to predict the participants’ PHQ-9 scores, yielding favorable regression accuracy. In Figure 3, we observe that MDD patients exhibit stable and lower conversion rates between different brain states compared to the other groups. This suggests that MDD patients have a reduced tendency for transitioning into brain activity states associated with stimuli evoking sadness. Consequently, it implies that sad emotional stimuli may not elicit as active brain responses in MDD patients. To explore the neurophysiological basis underlying this phenomenon, we computed the coherence between channels within each HMM state for the six experimental groups, as shown in Figure 4. Our analysis reveals a decreasing trend in frontal lobe connectivity in the MDD group when presented with grief stimulus materials. Furthermore, in Figure 5, we calculated the power spectral density for various frequency bands. Notably, we observed significant activation in the frontal and parietal regions in the theta and alpha frequency bands. This finding supports the association between the generation of sadness emotions and the frontal and parietal regions. Coupled with the reduced frontal connectivity observed in Figure 4, we can infer that the abnormal response of depressed patients to sadness stimuli is related to functional abnormalities in the frontal and parietal regions.

Our study demonstrates that the HMM-MAR model can serve as a decoding tool to analyze the neural dynamic processes of brain activity in cognitive tasks. Based on coherence analysis of brain connectivity patterns and power spectrum estimation results, significant differences in brain activation and connectivity patterns were observed between MDD patients and HC when presented with three emotional stimuli, which may suggest a risk for MDD in clinical practice. Furthermore, regression experiments revealed that the neural dynamic processes of MDD patients when presented with sad facial expressions exhibited Markovian properties and could be well characterized by the HMM-MAR model.

Regarding the differences in brain connectivity and activation between MDD and HC, there is ample evidence from previous studies that MDD may be a brain disorder characterized by changes in brain structure and function resulting from damage to certain brain tissues or abnormal neural circuits. The caudal middle frontal cortex (MFC) and the superior frontal gyrus (SFG) are structures of the frontal lobe, whose structural change has been considered to manifest anatomic abnormalities in MDD (du Boisgueheneuc et al., 2006), whose cortex volume saw a significant reduction with subjects suffered from long term depression (Lai et al., 2000). Besides, the degeneration in the temporal lobe was believed to be associated with the occurrence of first-episode MDD in a meta-analytical study (Bora et al., 2012). According to a morphology study on 77 adolescents aged 11–21 (Wang et al., 2011), the insula, the temporal pole, the parahippocampal, and the cingulate cortices were major components of the paralimbic zone (PZ), which circles around the medial and basal aspects of the cerebral hemispheres, plays a critical role in the regulation of emotional and neurovegetative functions that were disrupted in the core features of MDD. Moreover, accumulating pieces of evidence have revealed that MDD might originate in abnormal neural circuits. Consistent evidence widely distributed within the Default Mode Network (DMN) tissues was found in a functional MRI study that the MDD patients differed from controls during the performance of emotional tasks. Reduced functional connectivity and altered negative BOLD responses within the DMN were also reported in functional MRI studies (Grimm et al., 2009; Belleau et al., 2015; Yan et al., 2019). Therefore, the differences in connectivity and activation patterns between MDD and HC groups decoded by HMM-MAR can be supported by evidence in both brain structure and function. This suggests the scientific validity of using HMM-MAR for decoding dynamic neural processes.

The clinical impacts of the proposed method are in three folds. Firstly, the differential responses of the HC and MDD groups to sad facial stimuli, as analyzed using the HMM-MAR model, may represent a potential clinical diagnostic indicator for MDD. This approach could serve as a reference to determine whether an individual exhibits characteristics of MDD in its early stages. Furthermore, the property of Markovian dynamics observed in MDD patients when presented with sad facial emotional stimuli can provide guidance for the use of rTMS, tDCS, ECT, and other devices in clinical MDD treatment (Caldieraro and Cassano, 2019). This finding can assist clinical therapists to guide physical interventions at appropriate stages of treatment. Finally, a comprehensive analysis of the distinct transition patterns between the HC and MDD, as suggested by the proposed model, may facilitate the identification of unique differences in brain functional connectivity. This information could be of immense value in the development of novel medications for MDD.

The present study has the following limitations. Firstly, although the HMM-MAR model demonstrated its ability to capture the neural dynamic transition in the experiment, the order of HMM states was not constrained, leading to the HMM states obtained not being directly related to brain networks, such as the DMN, which is widely believed to have physiological and structural significance. Additionally, the experiment was conducted only on a dataset of 128-channel EEG task data from 53 subjects, and further studies should apply the model to more MDD datasets to validate the universality and the reproducibility of results.

To the best of our knowledge, this is the first study to employ HMM-MAR to decode the neuronal dynamic of MDD patients. Compared with the previous machine-learning or deep-learning-based studies, which regarded the decoding model as a black box, this work has its superiority in the spatiotemporal pattern interpretability by utilizing the Hidden Markov Model. It is also noteworthy that the proposed method highly coincides with the clinical routine. Therefore, integrating this decoding model into the clinical workflow will be beneficial to both MDD diagnosis and permit timely interventions based on the diagnosis.

## Data availability statement

The original contributions presented in this study are included in the article/supplementary material, further inquiries can be directed to the corresponding author.

## Author contributions

WJ contributed to the modeling, data analysis, and the draft of the manuscript. SD contributed to the drafting and statistical analysis. CX contributed to the interpretation and critical manuscript revision. HK contributed to the data preprocessing and parameter adjusting. HB contributed to the algorithms and critical manuscript revision. TZ, LM, and HL contributed to the data acquisition, design, and supervision. All authors have approved the final manuscript.

## Footnotes

1
http://modma.lzu.edu.cn/data/index/
